# Study of Singlet Oxygen Dynamics on Silicon Polymer Matrix

**DOI:** 10.1155/2019/2584686

**Published:** 2019-02-19

**Authors:** Jeong-Wook Hwang, Seung-Jin Jung, Il Heo, Hyun-A Son, Jong-Ho Kim, Kang-Kyun Wang, Yong-Rok Kim

**Affiliations:** Department of Chemistry, Yonsei University, Seoul 03722, Republic of Korea

## Abstract

We report a detailed analysis of singlet oxygen generated from the photofunctional polymer film (PFPF) matrix which is the silicone polymer film (PDMS) embedded with a photosensitizer. Activation and deactivation dynamics of singlet oxygen generated from PFPFs were investigated with time-resolved phosphorescence spectroscopy. The singlet oxygen generated from PFPFs was dissipated into three different regions of the polymer matrix; the inside (component A), the surface (component B), and the outside (component C). According to the deactivation dynamics of singlet oxygen in the polymer matrix, the components B and C are expected to be more important for various applications.

## 1. Introduction

Singlet oxygen, which is one of the reactive oxygen species, has been extensively studied due to the high reactivity and the selectivity in the chemical and/or biological reactions [1–3]. Especially, photo-induced singlet oxygen is very useful in the biological and environmental applications such as photodynamic therapy, photodynamic inactivation of bacteria, and disinfection of wastewater due to the controlled generation with the light [4–6]. Despite the advantages, the applications of singlet oxygen-induced by the photoexcited photosensitizer have some other problems with the additional pollution of the photosensitizers themselves and unintended reactions with nonspecific materials. To solve the problems in the applications, various photofunctional materials that included photosensitizers are developed by many researchers [7–12]. Among them, recently, the studies have reported the bactericidal and the cell proliferation effects with the photofunctional polymers that isolate photosensitizer insides of the polymer matrix, which may provide the solution of the problems previously described. Now the detailed qualitative characterization is required for the photofunctional polymers since the dynamics of singlet oxygen are expected to be different on the application interest. The dynamics of the core of polymer would be different from the interface of the photofunctional polymer. However, until now, effective singlet oxygen generation which is realistically influencing to the target material, from photofunctional polymer was not qualitatively analyzed. Also, a correlation between singlet oxygen and efficiency of cell proliferation, bactericidal effect, and decomposition effect of harmful material does not explicitly prove. Therefore, in order to exactly regulate the activation and/or inactivation of target materials using singlet oxygen, effective singlet oxygen generation from photofunctional polymer matrix should be qualitatively analyzed.

In this study, we report the qualification analysis of generated singlet oxygen from the photofunctional polymer matrix. In order to control the surface free energy of the polymer, the controlled thickness of photofunctional polymer films (PFPFs), which are PDMS embedded with TDCPP was fabricated by the spin coating method. Fabricated PFPFs were characterized by an optical microscope, XRD, absorption spectroscopy, and emission spectroscopy. Generation and deactivation dynamics of singlet oxygen from PFPFs confirmed with time-resolved phosphorescence spectroscopy.

## 2. Experimental Details

### 2.1. Materials

The photosensitizer, meso-tetra(o-dichlorophenyl)porphyrin (TDCPP), and 2,4,6-trichlorophenol (TCP) were purchased from Tokyo Chemical industry Inc. And polydimethylsiloxane (PDMS, KE-45) and all solvents were purchased from Shinetsu silicon Inc., and Merck co, respectively. The TDCPP solution was prepared at concentration of 9.7 × 10^−4^ M in mixture solvent (dichloromethane: ethanol = 95: 5 (vol%)). In order to fabricate the photofunctional polymer film (PFPF), PDMS (1 g) was mixed with prepared TDCPP solution (2 mL), and then it was magnetically stirred in the dark condition. After 20 min, the mixture was dropped on the glass plate in the spin coater (WON Co, LSC-101). And then it was kept in the vacuum oven at 25°C for 24 h in the dark condition. The film thickness was regulated by the rotation speed (100–1800 rpm) of the spin coater [13].

### 2.2. Characterizations of the Photofunctional Polymer Film

Optical microscope (Olympus, CKX-41, Japan) topography image was obtained to evaluate the thickness of fabricated PFPFs. Crystallographic characteristics of the surface of the film were investigated with an X-ray diffractometer (XRD, Rigaku, Ultima IV) working on Cu K_α_ radiation. Steady-state absorption and emission spectra were obtained with a UV-vis spectrophotometer (Hitachi, U-2800) and a spectrofluorometer (Hitachi, F-4500), respectively [14].

### 2.3. Detection of Singlet Oxygen Generation from the Photofunctional Polymer Film

In order to confirm the deactivated singlet oxygen component at the outside environmental condition of the polymer matrix, the generated singlet oxygen from PFPFs was directly measured with the phosphorescence signal from the de-excitation of singlet oxygen in the air, H_2_O, and D_2_O solution. The Nd-YAG (Continuum surelite II-10, 10 Hz, 7 ns FWHM pulse) pumped optical parametric oscillator (OPO) laser (Continuum OPO plus, 5 ns FWHM pulse) was utilized as an excitation source for detection of the time-resolved singlet oxygen phosphorescence [14]. The excitation wavelength for singlet oxygen generation was 511 nm. Phosphorescence signals were collected perpendicular to the excitation beam and detected with a monochromator (Optometrics LLC, mini-chrom04) and a NIR-PMT (Hamamatsu, H10330A). The signals were acquired by a 500 MHz digital oscilloscope (Agilent technology, DS07052A) and transferred to a computer for data analysis [14]. To check the singlet oxygen relaxation dynamic on the surface of PFPF, the layer of 2,4,6-trichlorophenol (TCP) molecules which are the singlet oxygen quencher, were formed on the surface of PFPFs. In order to control the concentration of the TCP layer, TCP solution of various concentrations (0.5–3.0 × 10^−7^ M) was dropped on PFPFs. And then, the TCP/PFPF films were dried in the vacuum oven at 25°C for 24 h in the dark condition. The singlet oxygen generation from PFPFs with TCP layer was measured with the same procedure as above. And, The most important factor of component C for influencing the target material was investigated by measuring the singlet oxygen lifetime in the presence of various concentrations of the TCP solution (1.0 × 10^−4^∼2.0 × 10^−3^ M).

## 3. Results and Discussion

**Figure 1:**
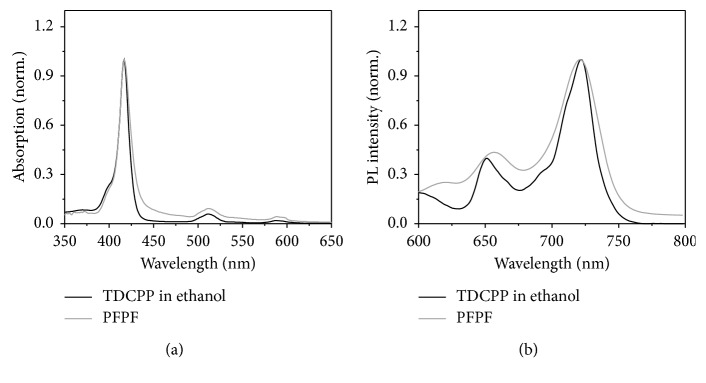
(a) Absorption and (b) emission spectra of TDCPP/ethanol and PFPF (*λ*_ex_ = 511 nm).

Thickness controlled PFPFs were fabricated by rotation speed controlled spin coating method.  Figure 1(a) shows the characteristics of TDCPP absorption bands: the soret band at 410 nm and the Q bands at 511 and 584 nm were nearly identical for TDCPP/ethanol and PFPFs. The fluorescence emission peaks of PFPFs (*λ*_ex_ = 511 nm) at 665 nm and 722 nm were also similar to those of TDCPP/ethanol (Figure 1(b)). The slightly red-shifted emission peaks of PFPF at 665 nm can be explained by the stabilization effect of the matrix [15]. The intensities of absorption and emission spectrum of PFPFs were increased linearly depending on the thickness of the films. Whereas, the shape and peaks position of absorption and emission had not been changed on various thickness of films. (not shown in the manuscript).

**Figure 2:**
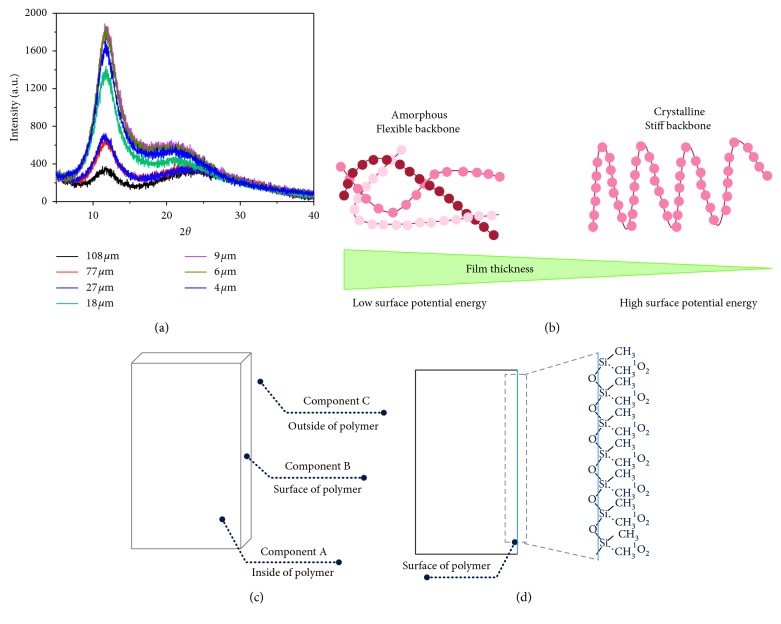
(a) The EDX spectra of PFPFs, (b) Schematic of polymer matrix structures (c) Schematics of singlet oxygen deactivation space, and (d) Cross section of the polymer matrix.

As shown in Figure 2, the strong Bragg reflection peaks of silicon (O–Si–O) (2*θ* = 12.0 and 21.5°) are marked by their Miller indices ((011) and (020)) from the previous report, which are the characteristic peaks of the tetragonal crystal lattice structure [16]. The crystallinity intensities have been determined for the different samples as shown in Table 1. As the thickness of PFPFs became thinner, intensities of peaks at 2*θ* of 12.0° were increased because crystallinity ratio of silicon (O–Si–O) on the surface of PFPFs was enhanced. And, the degree of the freedom of methyl group on the surface of PFPFs was reduced due to the fixation of total bonding angle between silicon atoms on the surface [17]. Also, structures of polymer surface were crystallized to follows the thickness of polymer became thinner (see the Figure 2(b)) [17]. Therefore, the surface free energy of thinner PFPF is increased, and the number of gas molecules which was trapped on the surface of PFPF was increased due to stabilize the surface free energy [18].

**Figure 3:**
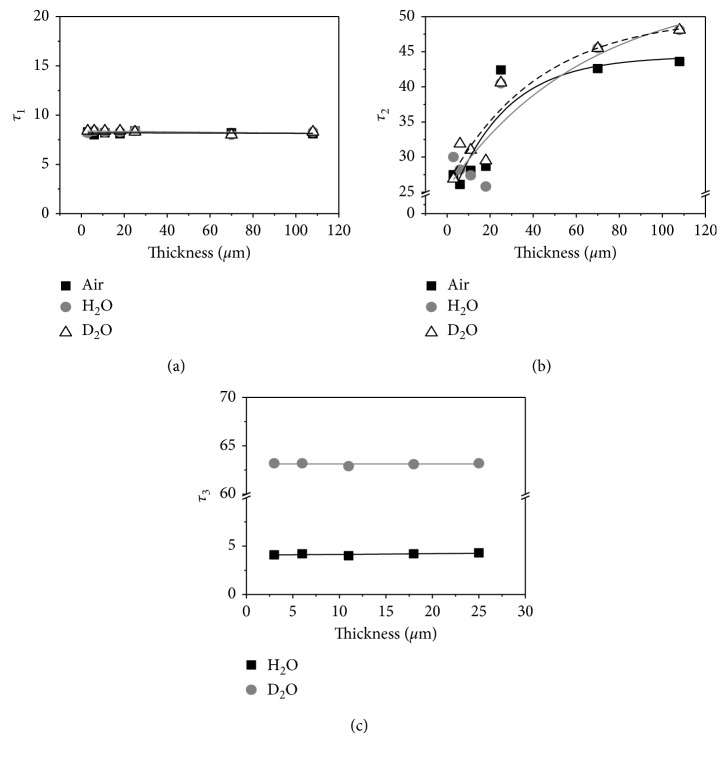
Plots for singlet oxygen lifetime of Component A, B, and C vs. the thickness of PFPFs in the H_2_O, D_2_O and air conditions.

The direct measurement method of singlet oxygen is the detection of the phosphorescence from the deactivation of singlet oxygen molecules induced by the photo-excited TDCPP within the silicone polymer. The excitation wavelength for singlet oxygen generation was 511 nm. The singlet oxygen phosphorescence signals from PFPFs were measured in air, H_2_O, and D_2_O condition at a detection wavelength of 1270 nm. The phosphorescence decay signals were fitted to a multi-exponential function. As shown in Table 2, component A of all samples shows a similar singlet oxygen lifetime of approximately 8 *μ*s in various environmental conditions. In the case of component B, the singlet oxygen lifetimes were changed by the increased surface free energy induced by the decreased thickness of the film. The increase of the surface free energy was reported to induce more adsorption of gas molecules for the energetical stabilization of the surface [19]. And, the increase of the surface adsorbed gas molecules such as oxygen, nitrogen, and dicarbon oxide, etc. can efficiently be experienced for the collisional quenching with the singlet oxygen on the surface of the film, which results the shorten lifetimes of the singlet oxygen on the surface [20, 21]. The singlet oxygen lifetimes of component C from all samples were fitted to 4 *μ*s in H_2_O solution and 62 *μ*s in D_2_O solution, respectively. These values have corresponded with a unique lifetime of singlet oxygen in H_2_O, D_2_O solution [22–25]. As shown in Figure 3, component A of all samples shows the same lifetime in various environmental conditions whereas the singlet oxygen lifetime of component B was depended on the thicknesses of PFPFs. And the singlet oxygen lifetime of component C was also depended on the outside environmental condition of the matrix. Generated singlet oxygen was deactivated in the inside (component A), the surface (component B), and out of the polymer matrix (component C). The singlet oxygen phosphorescence intensity of each component was estimated as a percentage scale, shown in Table 3.

**Figure 4:**
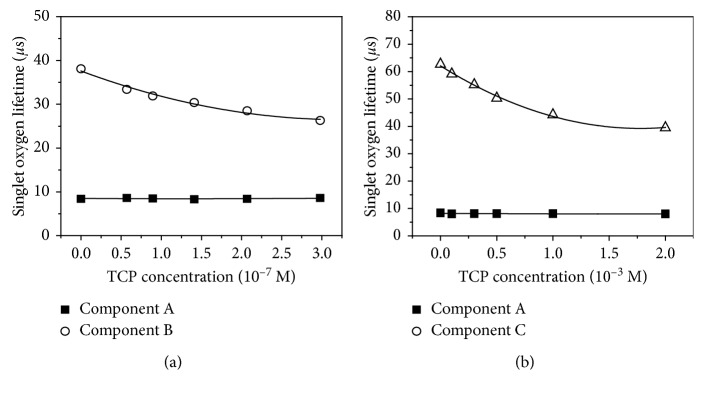
Plots of the singlet oxygen lifetime of (a) components A and B vs. TCP surface layer concentration, and (b) components A and C vs. TCP concentration in solution.

Furthermore, in order to check the application factor of components B and C for influencing the target material, the singlet oxygen phosphorescence signals from PFPF with the TCP surface layer, and within the TCP solution were measured, respectively. As shown in Figure 4(a), the singlet oxygen lifetime of component B was significantly decreased with increasing concentration of the surface adsorbed TCP.  Figure 4(b) represents that the singlet oxygen lifetime of component C was shorten depending on the increased concentration of the TCP in solution. On the other hand, the singlet oxygen lifetime of component A shows a similar value regardless of the TCP concentration. The results suggest that the generated singlet oxygen is all deactivated in the inside, on the surface, and the outside of the polymer matrix. The deactivated singlet oxygen on the surface, and the outside of the polymer matrix would be the critical components affecting the target materials.

## 4. Conclusion

We demonstrated the deactivation dynamics of singlet oxygen on the polymer matrix using the time-resolved singlet oxygen spectroscopic method. To control the surface free energy, the regulated thickness of photofunctional polymer films including photosensitizer was fabricated using the spin coating method. Fabricated PFPFs were characterized by an optical microscope, XRD, absorption spectroscopy, and emission spectroscopy. Generated singlet oxygen from PFPFs was deactivated on the inside, surface and outside of polymer matrix. And most of the singlet oxygen becomes extinct on the surface of PFPF. Among the generated singlet oxygen from PFPFs, component B (deactivated on the surface of the matrix) and C (deactivated on the out of matrix) only can influence to the target material. Therefore, quantization and qualification analysis of singlet oxygen generation from the photofunctional polymer are able to provide the fundamental information for the experimental design and the interpretation of experimental results in various application fields as photodynamic inactivation/activation of organ, decomposition of the environmental hormone, and singlet oxygen catalyst.

## Figures and Tables

**Table 1:** Thickness and crystallinity intensity of PFPFs.

Sample	Thickness of PFPFs (*μ*m)	Intensity at 12.0° of 2*θ* (a.u.)
S1	4	1543
S2	6	1771
S3	9	1837
S4	18	1275
S5	27	678
S6	77	660
S7	108	352

**Table 2:** Singlet oxygen lifetimes of PFPFs in the various environmental conditions (*χ*^2^ = 1.02 ± 0.03).

Singlet oxygen lifetime (*μ*s)									
Sample	Air			H_2_O			D_2_O		
	Component A τ_decay_	Component B τ_decay_	Component C τ_decay_	Component A τ_decay_	Component B τ_decay_	Component C τ_decay_	Component A τ_decay_	Component B Τ_decay_	Component C τ_decay_
S1	8.3 ± 0.4	27.5 ± 1.0	—	8.2 ± 0.1	30.0 ± 1.5	4.1 ± 0.1	8.4 ± 0.2	26.9 ± 1.2	63.2 ± 2.0
S2	8.0 ± 0.2	26.1 ± 0.4	—	8.4 ± 0.2	28.2 ± 0.4	4.2 ± 0.1	8.4 ± 0.3	31.9 ± 0.5	63.2 ± 2.4
S3	8.2 ± 0.2	28.1 ± 1.3	—	8.3 ± 0.1	27.4 ± 0.7	4.0 ± 0.1	8.4 ± 0.1	31.0 ± 0.3	62.9 ± 2.8
S4	8.1 ± 0.1	28.7 ± 0.8	—	8.3 ± 0.3	25.8 ± 0.8	4.2 ± 0.2	8.4 ± 0.1	29.5 ± 1.2	63.1 ± 2.4
S5	8.4 ± 0.1	42.4 ± 2.4	—	8.4 ± 0.3	40.5 ± 1.0	4.3 ± 0.1	8.3 ± 0.4	40.6 ± 2.0	63.2 ± 1.4
S6	8.2 ± 0.3	42.6 ± 1.1	—	8.0 ± 0.1	45.5 ± 1.4	—	8.0 ± 0.3	45.5 ± 1.3	—
S7	8.1 ± 0.1	43.6 ± 1.2	—	8.0 ± 0.3	48.1 ± 1.5	—	8.3 ± 0.2	48.1 ± 1.4	—

**Table 3:** Relative amplitudes of singlet oxygen phosphorescence from each component on PFPFs in the various environmental conditions.

Relative amount of singlet oxygen (%)									
Sample	Air			H_2_O			D_2_O		
	Component A	Component B	Component C	Component A	Component B	Component C	Component A	Component B	Component C
S1	2.1	97.9	—	3.1	85.2	11.7	2.3	86.7	11.0
S2	2.9	97.1	—	3.6	90.5	5.9	2.7	89.3	8.0
S3	4.9	95.1	—	5.1	91.3	3.6	4.3	92.6	3.1
S4	5.8	94.2	—	7.1	90.1	2.8	5.7	91.9	2.4
S5	12.8	87.2	—	11.4	86.1	2.5	13.8	84.2	2.0
S6	20.0	80.0	—	17.3	82.7	—	17.3	82.7	—
S7	26.9	73.1	—	21.4	78.6	—	21.4	78.6	—

## Data Availability

The figures used to support the findings of this study are included within the article.
